# Comparing sputum, nasopharyngeal swabs, and combined samples for respiratory bacterial detection using multiplex PCR

**DOI:** 10.1128/spectrum.02285-24

**Published:** 2025-01-30

**Authors:** Keun Ju Kim, Seung Gyu Yun, Myung-Hyun Nam, Chang Kyu Lee, Yunjung Cho

**Affiliations:** 1 Department of Laboratory Medicine, Korea University Anam Hospital, Korea University College of Medicine, Seoul, South Korea; Yale School of Public Health, New Haven, Connecticut, USA

**Keywords:** sputum, respiratory bacteria, combined samples, diagnosis, qPCR

## Abstract

Respiratory tract infections are major global health issues that require rapid and accurate diagnostic methods. Multiplex quantitative PCR (qPCR) is commonly used for pathogen detection in respiratory samples. However, the optimal specimen selection for detecting bacterial pathogens is not well-explored. We evaluated the detection rates of respiratory bacteria from nasopharyngeal swabs (NPS), sputum, and combined NPS and sputum samples using multiplex qPCR (Allplex PneumoBacter Assay, Seegene). Paired NPS and sputum samples from 219 patients with acute respiratory symptoms admitted to Korea University Anam Hospital were analyzed. qPCR was performed to detect seven respiratory bacteria: *Bordetella parapertussis, Bordetella pertussis, Chlamydophila pneumoniae, Haemophilus influenzae, Legionella pneumophila, Mycoplasma pneumoniae,* and *Streptococcus pneumoniae*. Combined NPS and sputum samples (*n* = 92) were created from 46 pairs of positive and 46 pairs of negative signals for NPS-alone and sputum-alone samples. Sputum samples (44.3%; 97/219) had a significantly higher positivity rate compared to NPS samples (21.0%; 46/219) (*P <* 0.001). The 92 combined samples identified a total of 65 bacterial nucleic acids. The detection rate for the combined samples was 86.2% (56/65), which was comparable to that of sputum alone (89.2%; 58/65) and higher than that of NPS samples (50.8%; 33/65). Combining NPS and sputum samples for PCR testing may offer an alternative for bacterial pathogen detection, providing rates comparable to those of sputum alone and greater sensitivity than that of NPS alone. This combined approach could be a cost-effective method to maximize diagnostic yield, reduce the need for multiple tests, and improve the management of respiratory infections.

**IMPORTANCE:**

This study offers important insights into refining diagnostic strategies for respiratory bacterial infections using multiplex PCR. This study finds that combining sputum and nasopharyngeal swabs into a single tube could serve as an effective alternative for detecting respiratory bacteria in adults with acute respiratory illness.

## INTRODUCTION

Respiratory tract infections, primarily caused by viruses and bacteria, are major contributors to mortality and morbidity worldwide (1). These infections typically present as acute illnesses with symptoms that develop rapidly, usually within hours to days after the initial infection. Common symptoms include fever, cough, sore throat, runny nose, shortness of breath, wheezing, and/or breathing difficulties (2). Rapid and accurate diagnosis of the causative agent is essential for effective treatment and preventing further transmission (1).

Microbiological culture is considered the gold standard for pathogen detection in laboratory diagnoses of microbial infections (3). Diagnosing respiratory bacterial illnesses generally involves culturing bacteria from the lower respiratory tract, such as sputum or bronchial alveolar lavage (4). However, interpreting sputum cultures can be challenging due to contamination from normal oropharyngeal flora and the presence of bacteria that are difficult to culture. As a result, sputum culture sensitivity is often suboptimal, especially in cases of pneumonia (5, 6).

Advanced molecular diagnostic technologies are poised to transform clinical microbiological diagnoses, offering increased speed and robustness (3). Nucleic acid amplification tests (NAATs), such as PCR, are now widely used in diagnostic laboratories to detect respiratory pathogens, largely due to the increased availability of commercial assays (7). NAATs offer several advantages over traditional culture methods, including rapid turnaround times, the ability to detect low levels of pathogens, independence from the viability of the target microorganism, minimal influence of antimicrobial therapy on diagnostic sensitivity, and potential for automation. Additionally, NAATs can provide supplementary information such as antimicrobial susceptibility data and strain typing (7). However, selecting the most appropriate specimen—from the upper or lower respiratory tract—remains challenging (3). Several studies have compared specimens and found similar but complementary results between different types of samples (8, 9). To achieve the highest diagnostic yield, assessing both types of specimens concurrently may be the most effective approach. Nonetheless, additional testing incurs increased costs, with multiplex PCR being particularly expensive. One of the primary objectives of diagnostic stewardship in clinical microbiology laboratories is to prevent unnecessary patient charges by optimizing testing practices (10). Clinical laboratories often receive multiple respiratory specimens from a single patient, and it is essential to manage these specimens efficiently to avoid redundant tests. Thus, previous studies demonstrated that combining sputum and nasopharyngeal swab (NPS) into a single tube is a cost-effective option for diagnosing viral infections via reverse transcriptase PCR, with minimal loss of sensitivity (11, 12). However, to our knowledge, no studies have evaluated the effectiveness of this approach for detecting bacterial nucleic acids. In this study, we aimed to compare the detection of respiratory bacteria using sputum, NPS, and combined sputum and NPS samples with a multiplex PCR assay.

## MATERIALS AND METHODS

### Study population

Participants were patients aged over 21 years admitted to Korea University Anam Hospital with acute respiratory symptoms from 1 October 2019 to 30 December 2019 prior to the COVID-19 pandemic. Only patients who could produce sputum were included. Those unable to generate sputum were excluded. Paired NPS and sputum specimens were collected from 219 patients.

### Clinical information

Data extracted from electronic medical records included age, sex, body mass index, underlying medical conditions, symptom duration, respiratory symptoms, results of sputum bacterial culture, prior antibiotic use before culture and PCR, results of respiratory viral infections via reverse-transcriptase PCR, and clinical outcomes (pneumonia diagnosis and mortality). Underlying medical conditions were categorized using the Charlson Comorbidity Index (CCI) scores (0, 1, and ≥2) (13).

### Respiratory sample collection and processing

During routine care, respiratory samples were collected. NPS samples were collected in 3 mL of universal transport medium (UTM; Copan Diagnostics). Each UTM tube contained three glass beads, which facilitated the release and dispersion of bacterial particles associated with host cells from patient materials during vortexing. After vortexing, 2 mL of UTM was transferred to a 2-mL tube and centrifuged at 13,000 × *g* for 1 minute. The supernatants were used for nucleic acid extraction, and the remaining sample volumes were preserved at −80°C for subsequent combined analysis.

Sputum specimens were collected in sterile containers and sent to the clinical microbiology laboratory. Sterile glass beads were used to liquefy the sputum during vortexing, following dilution with an equivalent volume of phosphate-buffered saline. Vortexing with glass beads facilitated the release of any cell-associated bacterial particles from patient materials. A 2-mL aliquot of the liquefied sputum was transferred to a tube and centrifuged similarly to the NPS samples. The supernatants were used for PCR analysis, while the remaining sputum samples were stored at −80°C.

### Quantitative PCR of respiratory bacteria

NPS and sputum samples were subjected to nucleic acid extraction using a MICROLAB STARlet IVD (Hamilton Robotics, Reno, NV) with the STARMag 96 × 4 universal cartridge kit (Seegene, Seoul, Korea). Following extraction, a multiplex quantitative PCR (qPCR) detection assay was performed using the Allplex PneumoBacter Assay (Seegene) according to the manufacturer’s instructions. NPS and sputum samples from each patient were tested in parallel for seven respiratory bacteria: *Bordetella parapertussis*, *Bordetella pertussis*, *Chlamydophila pneumoniae*, *Haemophilus influenzae*, *Legionella pneumophila*, *Mycoplasma pneumoniae*, and *Streptococcus pneumoniae*. A cycle threshold (Ct) value ≤42 was considered positive for bacterial detection. The assay was validated using both sputum and NPS specimens.

### Combining NPS and sputum into a single tube

After performing qPCR on paired NPS and sputum samples, combined samples (*n* = 92) for bacterial detection were prepared using 46 remnant NPS and sputum samples with positive bacterial signals and 46 with negative bacterial signals. The residual paired samples stored at −80°C were thawed and centrifuged at 13,000 × *g* for 1 minute. A 1-mL aliquot of each specimen supernatant was transferred into a new 2-mL tube and mixed by vortexing. The combined samples were then subjected to nucleic acid extraction and qPCR for the seven bacteria, as described previously.

### Statistical analysis

McNemar’s test was used to determine differences in positivity rates between paired NPS and sputum samples. Detection rate differences for each specimen type and variations in clinical characteristics were analyzed using the chi-squared test or Fisher’s exact test as appropriate. Unpaired comparisons of Ct values for *S. pneumoniae* and *H. influenzae* detected by NPS versus sputum samples were conducted using the Mann–Whitney U test following the Shapiro–Wilk normality test. Paired comparisons of Ct values for *S. pneumoniae* and *H. influenzae* detected by both NPS and sputum samples were conducted using the paired *t*-test following the normality test. Concordance between PCR and culture for *S. pneumoniae* and *H. influenzae* was assessed using overall concordance percentage and kappa. Comparisons of Ct values for bacteria detected in the paired NPS and sputum and the combined NPS–sputum samples were conducted using one-way ANOVA with Tukey’s correction or Kruskal–Wallis test with Dunn’s correction after the normality test. Statistical analyses were performed using IBM SPSS Statistics for Windows, version 26.0 (IBM Corp., Armonk, NY, USA) and GraphPad Prism version 10.2.3 for Windows (GraphPad Software, Boston, MA, USA). A *P* value < 0.05 was considered statistically significant.

## RESULTS

### Characteristics of study participants

The clinical characteristics of the study participants are summarized in Table 1. The median age was 73 years, and female participants constituted 45.7% (*n* = 100) of the cohort. Among the patients, 32 (14.6%) were ex-smokers, 21 (9.6%) were current smokers, and 25 (11.4%) had chronic lung disease. The cohort included a substantial number of patients with cancer (74, 33.8%) and 23 (10.5%) individuals with dementia. According to the CCI, 146 (66.7%) patients had preexisting medical conditions. The patients experienced respiratory symptoms for a median of 1 day before sampling (Table 1).

**TABLE 1:** Clinical characteristics of all patients and those with positive PCR diagnoses

Characteristics	*n* = 219	qPCR (NPS^*a*^ positive) *n* = 46	qPCR (sputum positive only) *n* = 54	*P* value
Age (years), median (interquartile range)	73.0 (61.5, 83.0)	75.0 (66.5, 84.0)	67.0 (60.75, 78.0)	0.061
Sex (no. (%) of females)	100 (45.7)	18 (39.1)	27 (50)	0.276
Body mass index, median (interquartile range)	23.35 (20.21, 25.42)	22.60 (19.03, 25.20)	23.74 (20.85, 26.85)	0.157
No. (%) of alcohol drinkers	57 (26.0)	14 (30.4)	13 (24.1)	0.475
No. (%) of current smokers	21 (9.6)	7 (15.2)	5 (9.3)	0.361
No. (%) of ex-smokers	32 (14.6)	4 (8.7)	9 (16.7)	0.237
No. (%) of patients with chronic lung disease	25 (11.4)	6 (13.0)	5 (9.3)	0.547
No. (%) of patients with a history of tuberculosis disease	11 (5.0)	2 (4.3)	3 (5.6)	1.000
No. (%) of patients with chronic cardiac disease	29 (13.2)	4 (8.7)	9 (9.3)	0.237
No. (%) of patients with dementia	23 (10.5)	11 (23.9)	4 (7.4)	0.021
No. (%) of patients with cancer	74 (33.8)	13 (28.3)	18 (33.3)	0.585
Charlson Comorbidity Index				
0	73 (33.3)	14 (30.4)	22 (40.7)	0.285
1–2	97 (44.3)	21 (45.7)	24 (44.4)	0.904
>2	49 (22.4)	11 (23.9)	8 (14.8)	0.248
Duration of symptoms, days prior to sampling, median (interquartile range)	1 (1, 4)	2 (1, 4)	1 (0, 5)	0.785
Cough	112 (51.1)	27 (58.7)	29 (53.7)	0.616
Febrile sense	100 (45.7)	21(45.7)	29 (53.7)	0.422
Sputum	97 (44.3)	19 (41.3)	19 (35.2)	0.530
Dyspnea	93 (42.5)	20 (31.5)	17 (43.5)	0.216
Fatigue	32 (14.6)	6 (13.0)	11 (20.4)	0.331
Myalgia	15 (6.8)	3 (6.5)	7 (13.0)	0.335
Rhinorrhea	14 (6.4)	5 (10.9)	2 (3.7)	0.243
Sore throat	6 (2.7)	4 (8.7)	0 (0)	–
No. of patients who used antibiotics before PCR	81 (37.0)	14 (30.4)	13 (24.1)	0.475
No. of patients with positive upper respiratory viral diagnosis using PCR	68 (31.1)	25 (54.3)	23 (42.6)	0.241
Clinical diagnosis of pneumonia	144 (65.8)	35 (76.1)	41 (75.9)	0.985
Mortality	32 (14.6)	10 (21.7)	6 (11.1)	0.148

^*a*^ NPS, nasopharyngeal swab; –, not applicable.

### Increased detection rates for respiratory bacteria using sputum

Bacterial nucleic acids were detected in paired samples from 100 of the 219 patients (45.7%) (Table 2). Of these patients, 43 (19.6%) had bacterial nucleic acids detected in both NPS and sputum samples, 54 (24.7%) had positive results only in sputum, and three (1.4%) were positive only in NPS samples (Table 2). Sputum samples had a significantly higher positivity rate (44.3%; 97/219) compared to NPS samples (21.0%; 46/219) (*P <* 0.001). In NPS samples, a single bacterium was identified in 38 cases (82.6%) and two bacteria in eight cases (17.4%). In sputum samples, a single bacterium was detected in 71 cases (73.2%) and two bacteria in 26 cases (26.8%). Overall, multispecies infections were not significantly associated with the specimen type (*P =* 0.217), nor were there differences in the list of etiologic agents detected between specimen types (Table S1).

**TABLE 2:** Comparison of qPCR results between NPS^*a*^ and sputum samples (*n* = 219)

	No. (%) of sputum samples		
	Positive	Negative	Total
No. (%) of NPS samples			
Positive	43 (19.6)	3 (1.4)	46 (21.0)
Negative	54 (24.7)	119 (54.3)	173 (79.0)
Total	97 (44.3)	122 (55.7)	219 (100)

^*a*^ NPS, nasopharyngeal swab.

In total, 128 bacterial signals were detected in paired samples from 100 patients (Table 3). The bacterial detection rates were 24.7% (54/219) for NPS qPCR and 55.7% (122/219) for sputum qPCR. The most frequently detected bacterium was *S. pneumoniae*, followed by *H. influenzae* (Table 3), with both identified more often in sputum than in NPS samples. Concordance between PCR and culture results was notably poor; among 219 patients, cultures identified only nine individuals with on-panel pathogens, including six cases of *S. pneumoniae* and three cases of *H. influenzae* (Table S2 and S3).

**TABLE 3:** Distribution of 128 bacteria among 100 patients with respiratory illness before combining the specimen types

	No. (%) of positive bacterial infection diagnoses				*P* value
Virus	Total	NPS^*a*^ and sputum	NPS only	Sputum only	
*Streptococcus pneumoniae*	68	25	4	39	<0.001
*Haemophilus influenzae*	50	16	2	32	<0.001
*Mycoplasma pneumoniae*	5	5	0	0	–
*Legionella pneumophila*	4	1	0	3	–
*Bordetella pertussis*	1	1	0	0	–
*Bordetella parapertussis*	0	0	0	0	–
*Chlamydophila pneumoniae*	0	0	0	0	–
Total	128	48 (37.5)	6 (4.7)	74 (57.8)	

^*a*^ NPS, nasopharyngeal swab; –, not applicable.

### Comparing Ct values between NPS and sputum samples

**Fig 1:**
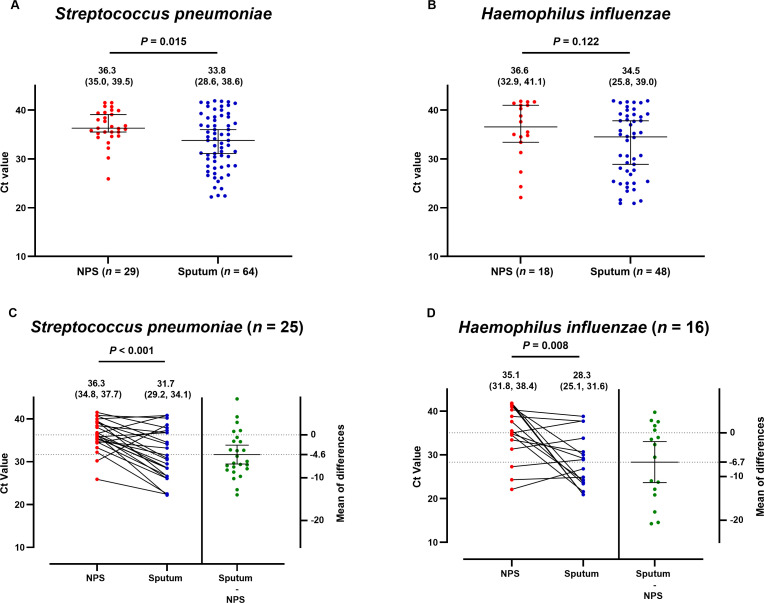
Comparison of PCR Ct values obtained from NPS and sputum samples for *Streptococcus pneumoniae* and *Haemophilus influenzae* detection. Unpaired Ct values of *S. pneumoniae* (**A**) and *H. influenzae* (**B**) detected in NPS and sputum samples were compared using the Mann–Whitney test. The median and interquartile range are shown. Paired Ct values of *S. pneumoniae* (**C**) and *H. influenzae* (**D**) detected in both NPS and sputum samples were compared using the paired Student’s *t*-test. The mean with 95% confidence interval (CI) is shown above the plot. The mean difference in Ct values between paired NPS and sputum samples is also indicated. The solid line with the error bar represents the mean with a 95% CI.

To assess bacterial load in different sample types, Ct values of the two most frequently detected pathogens, *S. pneumoniae* and *H. influenzae*, were compared. For *S. pneumoniae*, the median Ct values were lower in sputum than in NPS samples (*P =* 0.015) (Fig. 1A). This trend was also observed for *H. influenzae* (Fig. 1B), but without statistical significance (*P =* 0.122). Additionally, we compared the Ct values of bacteria in paired samples detected by NPS and sputum PCR. Sputum samples were associated with lower mean Ct values for both *S. pneumoniae* (*P* < 0.001; Fig. 1C) and *H. influenzae* (*P =* 0.008; Fig. 1D).

### Comparison of clinical characteristics

Overall, the clinical characteristics of patients were similar between those with positive NPS PCR and those with positive sputum PCR only, although dementia was more strongly associated with the NPS qPCR-positive group (Table 1). There was a trend toward a higher mortality rate in the group with positive NPS PCR results compared to the only sputum-positive groups, but rates of clinical diagnosis of pneumonia did not differ.

### Combined NPS and sputum samples

**Fig 2:**
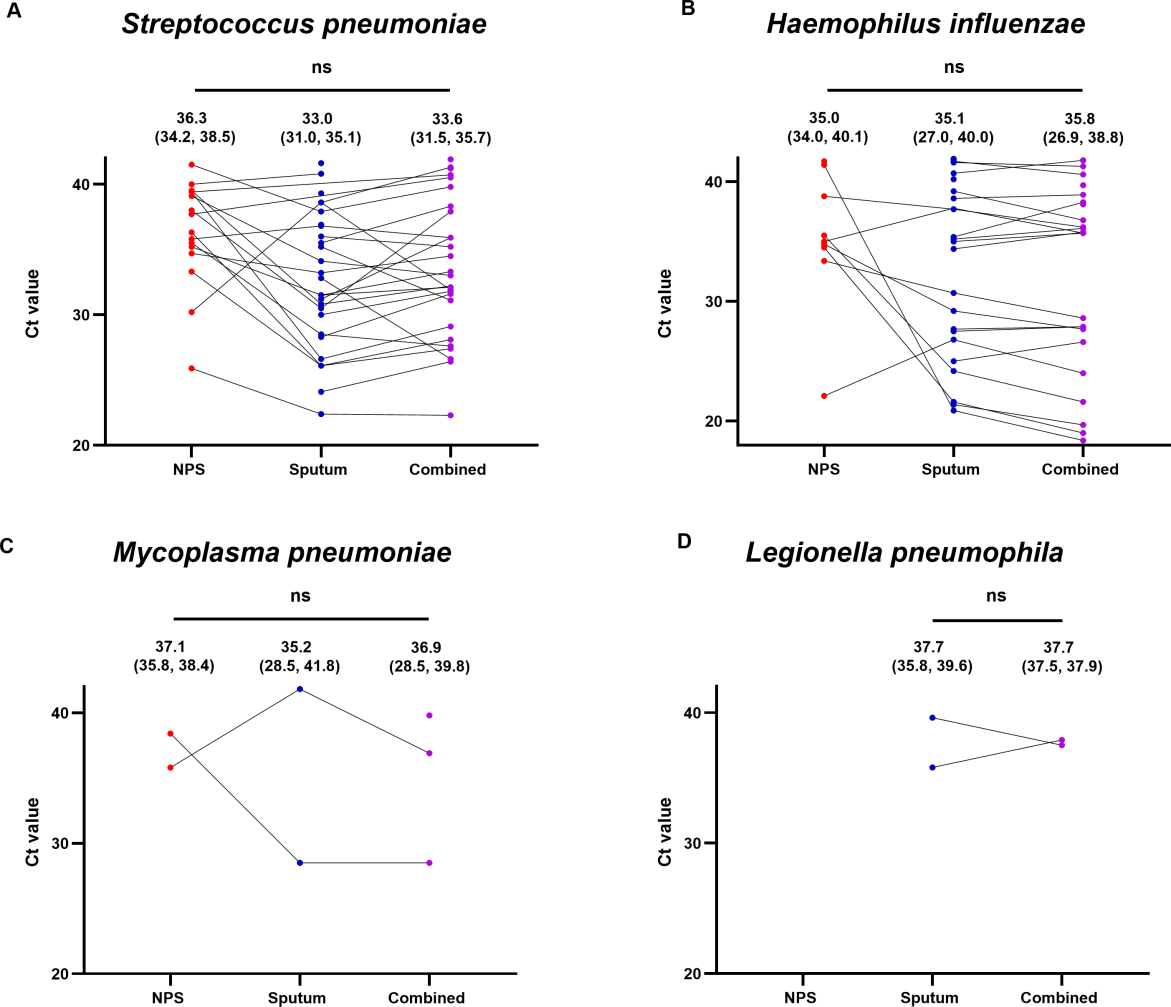
Comparison of PCR Ct values obtained from paired NPS and sputum samples before combining and from the combined NPS–sputum samples. The mean Ct values of *S. pneumoniae* (**A**) detected in each sample were compared using the one-way ANOVA test with Tukey’s correction after the Shapiro–Wilk normality test. The mean with 95% CI is shown above the plot. The median Ct values of *H. influenzae* (**B**), *M. pneumoniae* (**C**), and *L. pneumophila* (**D**) detected in each sample were compared using the nonparametric Kruskal–Wallis test with Dunn’s correction after the Shapiro–Wilk normality test. The median with interquartile range is shown above the plot. ns, nonsignificant.

**Fig 3:**
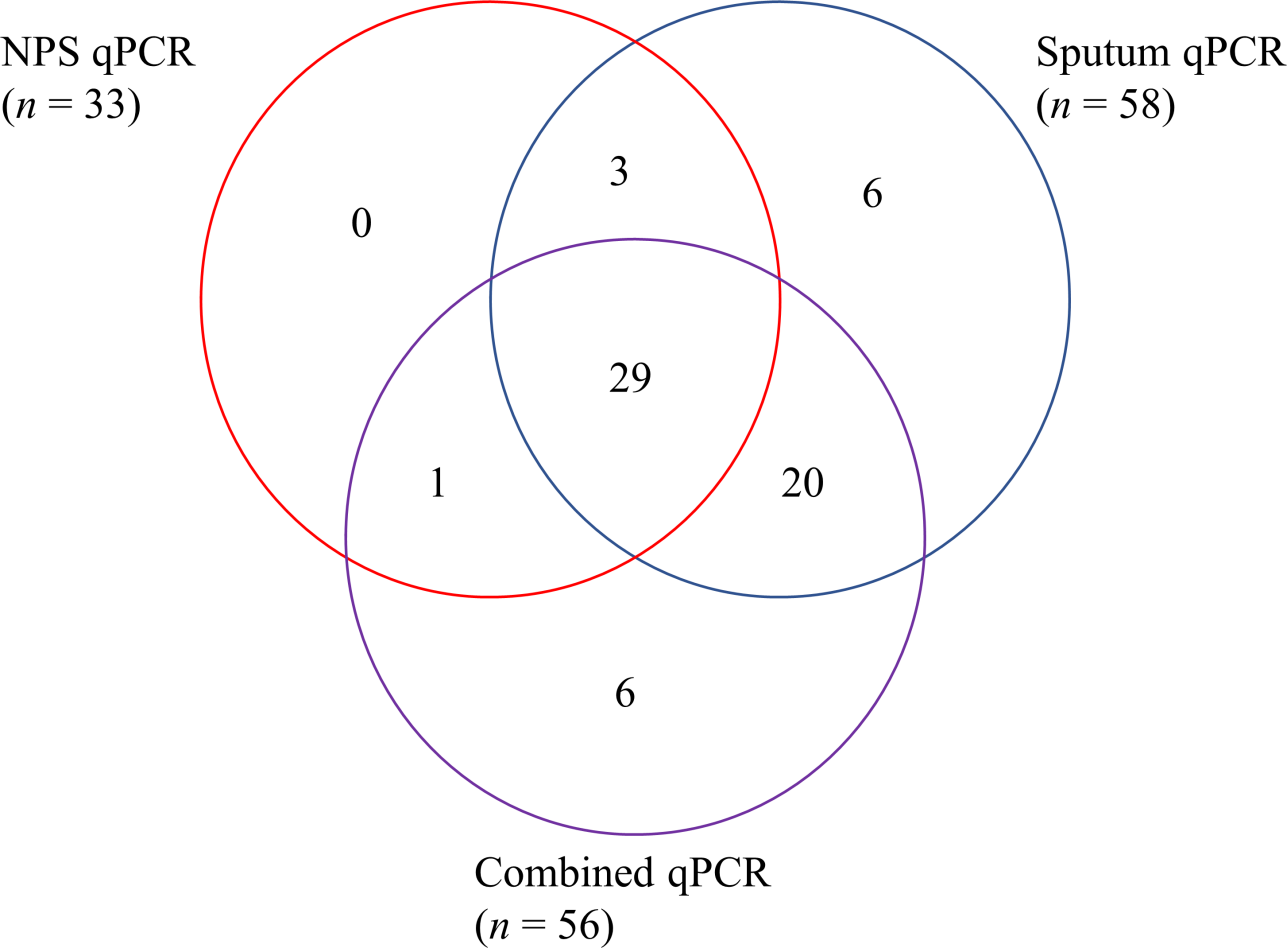
Venn diagram illustrating the distribution and overlap of positive tests for detecting bacterial nucleic acids. Each section of the diagram shows the number of distinct bacterial nucleic acids detected by each assay (total = 65).

For the combined NPS–sputum samples with a positive signal (*n* = 46), 22 (95.7%) of the 23 paired samples positive in both specimens were also positive in the combined NPS–sputum sample (Table 4). Of the 23 discordant paired samples, 20 (87.0%) were positive for the combined samples. Unexpectedly, a new *S. pneumoniae* signal, previously undetected, was identified in one of the 22 sputum-alone specimens after combination with the paired NPS. Similarly, among the combined samples with a negative signal (*n* = 46), five showed a new bacterial signal that was not detected before combining (Table 4). The Ct values for bacteria detected in paired NPS, sputum, and combined samples were analyzed, revealing no statistically significant differences across sample types (Fig. 2). Figure 3 shows the distribution of the positive bacterial nucleic acids. The combined samples retained most of the added bacterial DNA signals from the sputum (20/26), along with the six previously noted additional bacterial signals, despite losing three signals in the NPS samples after combining (Table 4; Fig. 3). The combined samples showed a detection rate of 86.2% (56/65), which was higher than the NPS detection rate of 50.8% (33/65) and comparable to the sputum detection rate of 89.2% (58/65) (Fig. 3).

**TABLE 4:** Overview of comparison results for paired NPS^*a*^ and sputum samples and combined NPS and sputum samples

Type of sample	No. of sample pairs tested before combining	No. of sample pairs (%) with a positive result after combining	No. of bacteria detected before combining	No. of bacteria detected (%) after combining
Total number of positives	46	42 (91.3)	59	51 (86.4)
NPS-positive alone	1	1 (100)	1	1 (100)
Sputum-positive alone	22	19 (86.4)	26	21 (80.8)^*b*^
Both NPS- and sputum-positive with a positive signal	23	22 (95.7)	32	29 (90.6)^*c*^
Both NPS- and sputum-negative	46	5	0	5^*d*^

^*a*^ NPS, nasopharyngeal swab.

^*b*^ Bacteria not detected after combining the specimens were three samples with *Streptococcus pneumoniae* (Ct values for each: 36.9, 39.3, and 41.6) and three with *Haemophilus influenzae* (40.2, 41.9, and 41.9). The bacterium newly detected after combining the specimens was *S. pneumoniae* (41.2).

^*c*^ Bacteria not detected were *S. pneumoniae* (Ct values for each: 39.3 and 40.0) and *H. influenzae* (41.7).

^*d*^ Bacteria newly detected were three samples with *H. influenzae* (Ct values for each: 39.7, 38.1, and 41.8), one with *S. pneumoniae* (41.9) and one with *Mycoplasma pneumoniae* (Ct value: 39.8).

## DISCUSSION

Rapid molecular diagnostics are highly effective tools for assessing and managing patients with suspected respiratory tract infections (14). Detection of respiratory pathogens is dependent on the type and quality of specimens collected, the timing of collection after the onset of clinical symptoms, the age of the patient, and transportation and storage of the sample before being tested in the laboratory. Ensuring a high-quality collection of the right specimens is essential for making an accurate and interpretable laboratory diagnosis (7). In this study, we compared the detection rates of respiratory bacterial pathogens between NPS and sputum samples, finding a significantly higher yield with sputum, consistent with previous studies (15, 16). Variations in panels, seasons, and patient groups could affect study outcomes. To maximize diagnostic sensitivity, testing both specimens simultaneously is recommended because each specimen still provides complementary results, as observed in the current and previous studies (9, 16). Nonetheless, owing to the high cost of syndromic molecular testing, additional tests can be burdensome for patients suspected of having respiratory tract infections. A more economical approach is to combine multiple specimens in a single tube for a single test. Previous studies have successfully evaluated this method for detection of respiratory viruses (10, 11). Our study is the first to demonstrate that this combined method retains most of the additional sputum results for bacterial pathogens, whereas a few low-burden bacterial signals were lost.

Respiratory tract infections are a major cause of morbidity and mortality worldwide, with lower airway infections being particularly significant (17). Opportunistic bacterial pathogens, which are responsible for most cases of pneumonia, can lead to both localized and invasive infections. Importantly, bacterial colonization in the upper respiratory tract often precedes these infections (17). The clinical presentation of respiratory infections ranges from mild symptoms, such as rhinorrhea and sore throat, to severe conditions, such as respiratory failure in immunocompromised individuals. Symptoms including cough, fever, wheezing, and shortness of breath are not pathogen-specific and may originate from either the upper or lower respiratory tract infections (14, 18, 19). The choice of the optimal specimen—upper respiratory tract samples (e.g., NPS) or lower respiratory tract samples (e.g., sputum)—depends on the causative agent and disease (20). For example, sputum is the preferred specimen for diagnosing pneumonia caused by *S. pneumoniae* or *H. influenzae*, using traditional staining and culture methods. Conversely, NPS is ideal for identifying pathogens such as *B. pertussis*, *C. pneumoniae*, or *M. pneumoniae* in cases of acute bronchitis, with NAATs serving as the primary diagnostic approach. Diagnostic stewardship for infectious disease testing requires a multidisciplinary approach to optimize test selection, performance, interpretation, and patient treatment (21). With the increased availability and complexity of NAATs, there is also an increased need for collaborative approaches to optimize test use to promote positive impacts on patient care, while mitigating potential negative impacts or resource waste. From a diagnostic stewardship perspective, clinical microbiology laboratories play a crucial role in implementing efficient testing strategies to optimize specimen management, reduce unnecessary patient bills, and improve healthcare resource utilization (10). In our study, patients presenting with respiratory symptoms to a tertiary care hospital provided multiple respiratory specimens simultaneously. The rationale for this study was to provide insights into improving these stewardship practices by combining two different respiratory specimens into a single tube for a single PCR testing.

The nasopharynx is a complex and dynamic environment, where organisms capable of causing acute respiratory tract infection, such as *S. pneumoniae*, *H. influenzae*, *Staphylococcus aureus*, and *Moraxella catarrhalis*, may also exist as colonizers (22). Asymptomatic nasopharyngeal carriage often precedes lower respiratory tract infections and contributes to transmission. For bacteria not typically classified as nasopharyngeal colonizers, including *C. pneumoniae*, *L. pneumophila*, *M. pneumoniae*, and *B. pertussis*, a positive PCR result in symptomatic patients indicates the presence of a pathogen (22). In cases where potential colonizers, such as *S. pneumoniae* or *H. influenzae*, are detected, bacterial density can aid in distinguishing colonization from infection. Additionally, clinical and radiological findings related to specific symptoms should be used in conjunction with microbiologic results to differentiate true pathogens from colonizers (23). In the human upper respiratory tract, *Haemophilus* species account for around 10% of the bacterial population. *H. influenzae* acts as an opportunistic pathogen, typically colonizing the pharyngeal mucosa in humans without causing noticeable symptoms (24). This bacterium may potentially travel to the lower respiratory system through the nasopharynx, which acts as a reservoir for infection (25). Similarly, *S. pneumoniae*, a commensal bacterium, frequently inhabits the upper respiratory tract. However, colonization can progress to pneumococcal disease, manifesting in conditions such as otitis media, pneumonia, bacteremia, sepsis, and sometimes meningitis (26). This makes obtaining samples directly from the infection site particularly challenging (27). Although the sensitivity and specificity of molecular testing using sputum can be compromised by contamination with colonizers on the upper respiratory tract, it is generally accepted as the best nonsterile respiratory specimen for the recovery of the typical respiratory bacterial pathogens, *S. pneumoniae* and *H. influenzae* (28). However, laboratories must implement procedures to screen sputum samples for quality, excluding those heavily contaminated with oropharyngeal microbiota and unrepresentative of deep expectoration (20). Demars et al*.* (15) and Wolff et al*.* (16) reported that sputum is superior to the upper respiratory tract specimens for the detection of *S. pneumoniae* and *H. influenzae* both for adults and children, while Nyawanda et al*.* (9) reported that there is no clear advantage in using sputum specimens over nasopharyngeal/oropharyngeal swabs to investigate the etiology of acute lower respiratory infection using molecular diagnostics. In our study, sputum was associated with a higher detection rate of the two common respiratory bacterial pathogens compared to NPS. Many bacterial pathogens detected in respiratory specimens could reflect carriage, being part of the normal microbiome, and not necessarily associated with the disease process (9). For instance, in six cases in our study, *S. pneumoniae* or *H. influenzae* was present in NPS but absent in paired sputum samples, suggesting these bacteria might be common colonizers and not necessarily associated with the patients' clinical symptoms. Therefore, their presence, particularly when not detected in sputum, should not be assumed to indicate acute respiratory illness. However, when both NPS and sputum samples are positive, the higher bacterial load in sputum implies that these pathogens are less likely to be normal flora. *H. influenzae* species is categorized into seven groups, comprising six that display unique serotypes of polysaccharide capsules (a–f) and one unencapsulated group known as nontypeable *H. influenzae* (29). There are also at least 98 serotypes of *S. pneumoniae* that can lead to noninvasive illnesses (i.e., pneumonia, sinusitis, and otitis media) as well as invasive conditions (i.e., meningitis and bacteraemia) (30). Consequently, in cases where both NPS and sputum samples test positive in our study, examining the serotypes of *S. pneumoniae* and *H. influenzae* in both sample types could offer valuable insights into the relationship between bacterial carriage and infection. However, our study did not address this aspect, which represents a limitation. Further investigation is required to clarify this issue. Therefore, it is essential to correlate molecular diagnostic results with bacterial cultures and the clinical symptoms of patients to draw accurate conclusions.

In our study, compared to sputum culture, the use of NAAT led to an increase in the detection of *S. pneumoniae* and *H. influenzae*, which is consistent with previous findings (31, 32). The increase in positive results reported by molecular testing is not unexpected due to the detection of both viable and nonviable organisms, as well as the detection of low-abundance targets and those not recovered in culture due to fastidious growth characteristics (31). Moreover, some specimens were collected after starting antibiotic therapy, which was also the case for some of the participants in our study. One particular survival strategy in bacteria is the ability to enter a viable but nonculturable (VBNC) state that permits endurance to unfavorable environmental conditions such as the prior use of antibiotics (33). It is known that *H. influenzae* and *S. pneumoniae* enter the VBNC state when they are surrounded by harsh conditions (33–35). This state could also lead to a higher rate of detection by molecular testing compared to that by culture. Therefore, the interpretation and potential significance of these results require special attention to determine the impact of reporting on patient management (31). In addition, it is crucial that sputum specimens be representative of lower respiratory secretions to be of value for microbiological diagnosis (28), which could affect sputum culture interpretations and recovery rates.

**Fig 4:**
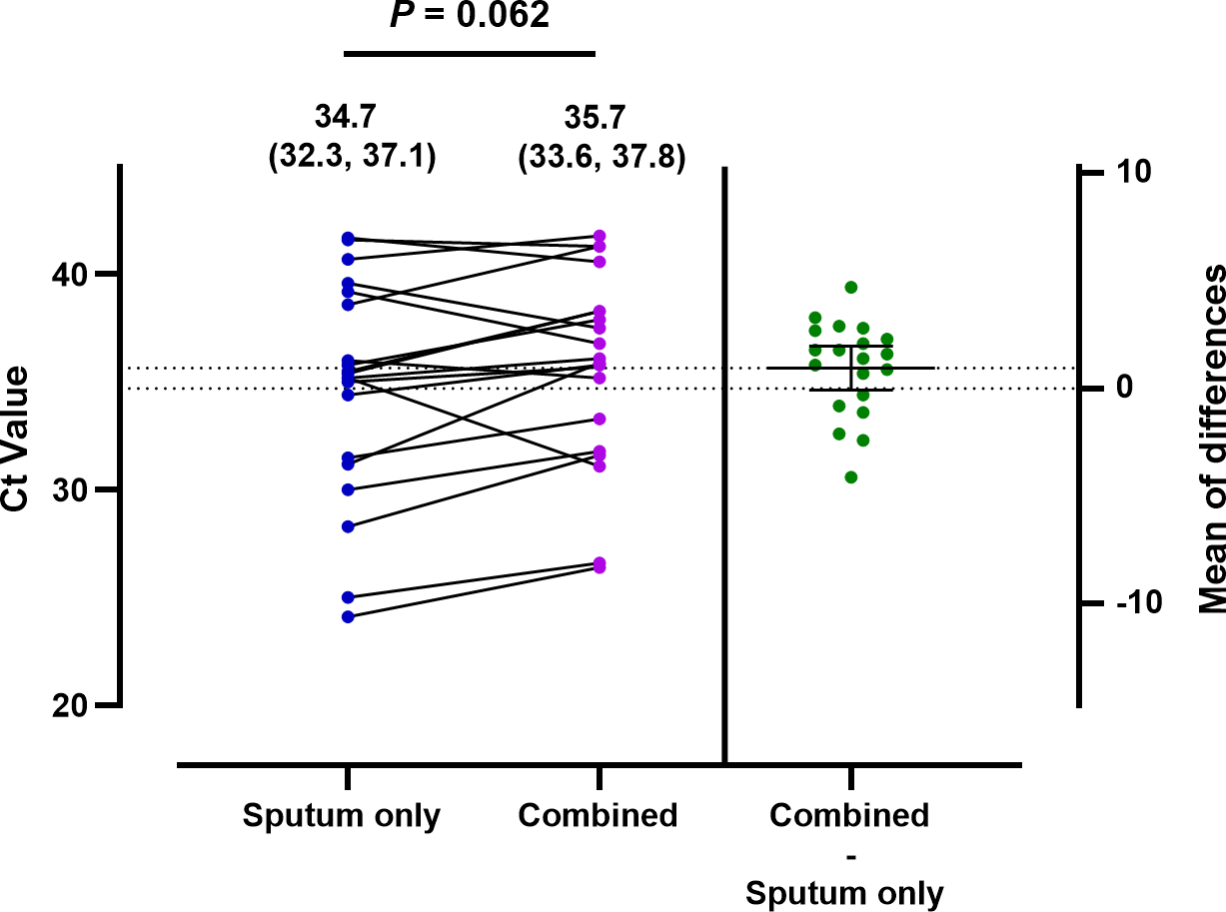
Comparison of Ct values of bacterial DNA detected by sputum only and after combining sputum and NPS specimens. The paired Ct values of all bacteria detected before and after combining specimens were compared using the paired Student’s *t*-test. The mean with 95% confidence interval (CI) is shown above the plot. The mean difference in Ct values between the two specimen types is also indicated. The solid line with the error bar represents the mean with a 95% CI.

Managing healthcare costs has become an economic necessity (36). Diagnostic testing expenses typically account for over 10% of total healthcare expenditures, and this proportion is increasing rapidly (37). Molecular diagnostic testing, in particular, may contribute to significant cost escalations (38). Furthermore, certain diagnostic tests are frequently misused or overused (39, 40). As the healthcare landscape shifts from a fee-for-service model to managed care or bundled payment systems that prioritize efficient resource use, there is an urgent need to improve diagnostic efficiency and minimize costs (12). Clinical laboratories often have multiple specimens submitted from the same patient, which adds to the cost. The significance of this study is that we investigated the performance of combined sputum and upper respiratory tract specimens in a single tube for diagnosing respiratory bacterial infections. Our findings suggest that the combined method may provide another option for detecting respiratory bacterial pathogens because each NPS and sputum sample provided complementary results. Interestingly, despite the dilution of raw specimens, we observed six previously undetected bacterial signals after combining. While this seems improbable, given the twofold dilution, the high viscosity of sputum may lead to an uneven distribution of bacteria, even after homogenization. To test this hypothesis, we compared the Ct values of the bacterial signals detected only in sputum before and after combining (Fig. 4). Six signals showed reduced Ct values after combining, suggesting that the new bacterial signals detected might be due to the heterogeneous nature of sputum. The freeze–thaw step is a recognized method for cell lysis and homogenization due to the mechanical disruption caused by ice crystal formation during freezing and their subsequent melting during thawing (41). In the combined samples, the additional freeze–thaw process may have improved bacterial DNA recovery by lysing the thick cell wall of gram-positive bacteria and/or homogenizing viscous sputum, thereby facilitating the release of bacterial DNA. Conversely, the nine bacterial signals with high Ct values detected before combining were lost after combining (Table 4; Fig. 2), possibly due to dilution. Another explanation could be the additional freeze–thaw cycle. Freezing can cause DNA strand breaks in cells, and it has been reported that freezing and thawing may reduce DNA extraction yield (42). Overall, this study showed that the combined method has a detection rate comparable to that of sputum alone, with minimal compromise in diagnostic sensitivity. The findings may help clinicians manage patients with acute respiratory symptoms by guiding the selection of sample types and optimizing testing strategies to accommodate patients’ financial situations. This approach can reduce the number of tests while improving diagnostic decisions.

The study had several limitations. First, the quality of sputum samples was not evaluated. All sputum specimens underwent PCR testing, and contamination from upper respiratory secretions may have been present. This contamination could have contributed to higher detection rates of *S. pneumoniae* and *H. influenzae* by PCR compared to culture methods. However, this is considered unlikely due to significantly lower Ct values for sputum compared to NPS samples for these pathogens. Another limitation is the absence of certain targets in the PCR test; specifically, *B. parapertussis* and *C. pneumoniae* were not included, and the detection of other pathogens such as *M. pneumoniae*, *L. pneumophila*, and *B. pertussis* was limited compared to that of *S. pneumoniae* and *H. influenzae*. The prevalence of etiological agents responsible for bacterial respiratory tract infections varies by season, population, and region. Additionally, the study was conducted among adult patients who were able to produce sputum at a single center, excluding populations such as young children and critically ill patients who may have difficulty expectorating. This selection could have introduced bias and reduced the generalizability of the results as these populations may present with distinct pathogen profiles or disease severities. For instance, *M. pneumoniae* is a leading cause of respiratory tract infections in humans, particularly in children and adolescents. It has become a prevalent pathogen in pediatric community-acquired pneumonia, accounting for 10%–40% of cases (43). Similarly, pertussis (whooping cough) caused by *B. pertussis* has re-emerged as a public health concern in several developed countries, despite high vaccination coverage among infants and young children (44). Among adult patients, *L. pneumophila* is frequently observed as a causative agent of severe community-acquired pneumonia, often alongside *S. pneumoniae* (45). Although glass beads were used to aid in the release of intracellular bacteria from patient materials, the non-detection of *C. pneumoniae*, an obligate intracellular pathogen, and the low detection rate of *L. pneumophila*, a facultative intracellular bacterium, suggest that vortexing with glass beads may not be sufficient for effectively extracting intracellular pathogens from host cells. Incorporating enhanced lysis protocols in the extraction process could improve the detection rates for intracellular bacteria. Therefore, the findings of this study should be interpreted with caution. Future studies incorporating alternative sampling and extraction methods and broader patient populations, including children and critically ill individuals, are necessary to validate these results and improve their applicability.

In conclusion, qPCR of sputum specimens showed a significantly increased detection rate of respiratory bacterial nucleic acids in adult patients admitted to the hospital, compared to NPS samples. Importantly, the combination of NPS and sputum samples maintained the detection rate achieved with sputum alone. These findings enhance our understanding of the optimal specimens for detecting respiratory bacterial pathogens using PCR.

## Data Availability

In the article or as supplementary information, all data pertinent to the study are included.

## SUPPLEMENTAL MATERIAL

Supplemental tables spectrum.02285-24-s0001.docx Tables S1 to S3.

ASM does not own the copyrights to Supplemental Material that may be linked to, or accessed through, an article. The authors have granted ASM a non-exclusive, world-wide license to publish the Supplemental Material files. Please contact the corresponding author directly for reuse.
