# Supplementary material for: Comparing sputum, nasopharyngeal swabs, and combined samples for respiratory bacterial detection using multiplex PCR
Source: Microbiol Spectr. 2025 Jan 30;13(3):e02285-24. doi: 10.1128/spectrum.02285-24 (PMC11878093; doi:10.1128/spectrum.02285-24)
Supplement: Supplemental tables — Tables S1 to S3. [file spectrum.02285-24-s0001.docx]

Table S1 Distribution of bacteria in patients with co-infections

| Multispecies bacteria detected | NPS samples (no. (%)) | Sputum samples (no. (%)) | *P* value |
| --- | --- | --- | --- |
| *Streptococcus pneumoniae* + *Haemophilus influenzae* | 7 (15.2) | 22 (22.7) | 0.300 |
| *Streptococcus pneumoniae* + *Legionella pneumophila* | 0 (0) | 3 (3.1) | Not applicable |
| *Legionella pneumophila + Haemophilus influenzae* | 1 (2.2) | 1 (1.0) | 0.541 |

Table S2 Concordance between culture and PCR analysis for *Streptococcus pneumoniae* diagnosis

| No (%). of culture samples | No (%). of PCR samples | | |  |  |
| --- | --- | --- | --- | --- | --- |
|  | Positive | Negative | Total | Percent overall agreement (95% CI^a^) | Kappa (95% CI) |
| Positive | 6 (2.7) | 0 (0) | 6 (2.7) | 73.5 (67.3, 78.9) | 0.128 (0.034, 0.222) |
| Negative | 58 (26.5) | 155 (70.8) | 213 (97.3) |  |  |
| Total | 64 (29.2) | 155 (70.8) | 219 (100) |  |  |

^a^CI, confidence interval.

Table S3 Concordance between culture and PCR analysis for *Haemophilus influenzae* diagnosis

| No (%). of culture samples | No (%). of PCR samples | | |  |  |
| --- | --- | --- | --- | --- | --- |
|  | Positive | Negative | Total | Percent overall agreement (95% CI^a^) | Kappa (95% CI) |
| Positive | 3 (1.4) | 0 (0) | 3 (1.4) | 79.5 (73.6, 84.3) | 0.094 (-0.006, 0.) |
| Negative | 45 (20.5) | 171 (78.1) | 216 (98.6) |  |  |
| Total | 48 (21.9) | 171 (78.1) | 219 (100) |  |  |

^a^CI, confidence interval.
